# Newly Discovered Atmospheric Oxidant Contributes to Climate Change, Sulfuric Acid Production

**DOI:** 10.1289/ehp.120-a422

**Published:** 2012-11-01

**Authors:** Nate Seltenrich

**Affiliations:** Nate Seltenrich covers science and the environment from Oakland, CA. His work has appeared in *High Country News*, *Sierra*, *Earth Island Journal*, the *San Francisco Chronicle*, and other local and national publications.

One scientist’s “noise” can be another’s data. Lee Mauldin, a researcher in the University of Colorado Boulder’s Department of Atmospheric and Oceanic Sciences, found both in the air above Finland’s boreal forest, where background levels of sulfuric acid (H_2_SO_4_) led to the discovery of a new atmospheric oxidant.^1^

Atmospheric H_2_SO_4_ contributes to acid rain and the creation of aerosols (particles suspended in air), which are associated with asthma, decreased lung function, and other ailments. Aerosols also play a complex role in climate science by affecting cloud formation and by scattering or absorbing sunlight.^2^

H_2_SO_4_ is produced by the oxidation of sulfur dioxide (SO_2_) released from volcanoes and various industrial processes. Atmospheric scientists have presumed that the rate at which SO_2_ is converted to H_2_SO_4_ depends on how much hydroxyl radical (OH)—which oxidizes SO_2_ in the presence of sunlight—there is.^3^ However, field measurements taken by Mauldin’s team at the Station for Measuring Ecosystem–Atmosphere Relations (SMEAR II) in Juupajoki, Finland, revealed evening concentrations of H_2_SO_4_ up to 10^6^ molecules/cm^–3^, which was at least 10 times higher than expected, according to Mauldin. This suggested another source for H_2_SO_4_ production; the investigators called this unknown source “X.”^1^

**Figure:**
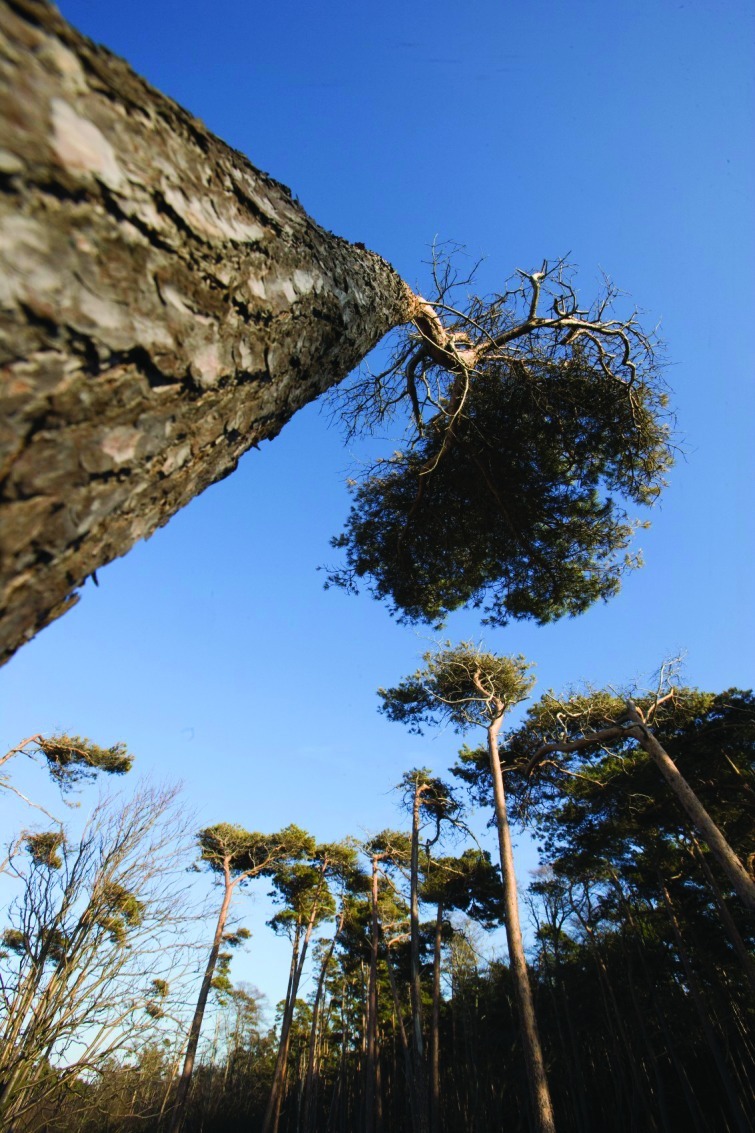
A new oxidant dubbed “X” was discovered in a Finnish forest dominated by Scotch pine (Pinus sylvestris). These and other trees emit hydrocarbons called alkenes that react with ozone to form X. © Dietmar Nill/ Foto Natura/Minden Pictures/Corbis

In previous experiments, Mauldin’s team calculated the concentration of OH in ambient air samples by adding SO_2_, then measuring the resulting H_2_SO_4_ levels using chemical ionization mass spectrometry. In order to isolate the H_2_SO_4_ attributable to the reaction between OH and SO_2_ and not to the presence of X, the researchers repeated the experiment with an OH scavenger and subtracted the background H_2_SO_4_.

The researchers found that X did not exhibit a diurnal cycle like OH, and that it typically exceeded OH in total concentration—further evidence of a link to the high H_2_SO_4_ levels measured at night. “As soon as I realized that we were observing a new oxidant, the light clicked on all over the place,” Mauldin said. “With anything that can produce sulfuric acid, if you can come up with something that occurs on a daily basis, or in this case 24/7, it can affect all sorts of things, including climate and human health.”

In the Great Smoky Mountains of the eastern United States, alkenes emitted by the trees react with ozone to produce OH that contributes to the formation of H_2_SO_4_ and to aerosols; this lends the range its characteristic haze.^2^ Suspecting that alkenes emitted by Finland’s boreal forest could be responsible for the formation of X, the researchers placed bare spruce, pine, and birch branches near the test instrument’s inlet. Even with the OH scavenger, H_2_SO_4_ concentrations rose by 10 to 100 times in the presence of the spruce and pine (but not birch) branches.^1^

The authors note that X is likely a stabilized Criegee intermediate—a type of carbonyl oxide formed when alkenes react with ozone that is known to oxidize SO_2_.^4^ However, another ozonolysis derivative may also be responsible.^1^

This uncertainty doesn’t diminish the importance of the findings, says Ron Cohen, director of the Berkeley Atmospheric Science Center at the University of California, Berkeley. “There’s a significant particle problem in the southeastern United States and parts of the Central Valley of California,” he says. “This understanding of this oxidation sequence is going to get us a long way toward understanding the aerosol [pollution] in those regions.”

## References

[1] Mauldin RL III A new atmospherically relevant oxidant of sulphur dioxide. Nature 488 7410 193 196 2012http://dx.doi.org/10.1038/nature11278 22874964

[2] Aerosols: Tiny Particles, Big Impact [website]. Greenbelt, MD:NASA Earth Observatory, EOS Project Science Office, National Aeronautics and Space Administration (updated 2 Nov 2010). Available: http://earthobservatory.nasa.gov/Features/Aerosols [accessed 11 Oct 2012].

[3] Heard D Atmospheric chemistry: the X factor. Comment on article by Mauldin III RL, et al. Nature 488 7410 193 196 2012http://dx.doi.org/10.1038/nature11278 22874960

[4] Welz O Direct kinetic measurements of Criegee intermediate (CH_2_OO) formed by reaction of CH_2_I with O_2_. Science 335 6065 204 207 2012http://dx.doi.org/10.1126/science.1213229 22246773

